# Cyclophosphamide-induced Atrial Fibrillation With Rapid Ventricular Rate

**DOI:** 10.7759/cureus.2633

**Published:** 2018-05-16

**Authors:** Komal Ejaz, Muhammad A Raza, Shahram Maroof, Muhammad W Haider

**Affiliations:** 1 Medicine/Sheikh Zayed Hospital, University of Health Sciences, Rahim Yar Khan, PAK; 2 Internal Medicine, Jinnah Hospital Lahore (jhl)/allama Iqbal Medical College (aimc), Lahore, PAK; 3 Internal Medicine/John D Dingell VA Medical Center, Wayne State University, Detroit, USA

**Keywords:** cyclophosphamide induced atrial fibrillation, cyclophosphamide induced cardiotoxicity

## Abstract

Cyclophosphamide (CYA), also known as cytophosphane, is a medication used as a chemotherapeutic agent and immune suppressor.Its common adverse effects include nausea, vomiting, diarrhea, bone marrow suppression, hemorrhagic cystitis, alopecia, lethargy, and cardiotoxicity. Cyclophosphamide-related cardiac toxicity is not uncommon and causes potentially serious complications in patients. In this review, we present a case of a 65-years-old patient who developed atrial fibrillation with rapid ventricular rate (RVR) after receiving a single dose of CYA. In this case, the advanced age of the patient, pre-treatment with prednisone, and renal insufficiency most likely predisposed the patient to CYA-induced cardiac toxicity. A relevant literature review was also conducted to determine the pathogenesis, risk factors, and spectrum of CYA-induced cardiac toxicity.

## Introduction

Cyclophosphamide (CYA) is a widely used chemotherapeutic and immuno-suppressant drug. As a chemotherapeutic agent, it is used to treat leukemia, lymphoma, multiple myeloma, breast cancer, ovarian cancer, lung cancer, neuroblastoma, and sarcoma. As an immune suppressor, it is used in nephrotic syndrome, autoimmune disorders, and following organ transplant. It acts via suppression of both humoral and cell-mediated immunity. Cardiotoxicity has been reported with higher doses of cyclophosphamide but here we present a case in which the patient experienced adverse cardiac event after receiving a single dose which was well below the threshold level.

## Case presentation

A 65-year-old African American male with past medical history of hypertension and chronic obstructive pulmonary disease (COPD) presented to the emergency department with the complaints of hemoptysis, hematuria, and mild midsternal chest pain for one week. He described his sputum as intermittent, mild/moderate in volume, and comprised of mucus mixed with blood. Chest pain was described as mild, pressure-like, non-progressive, non-radiating, worse with exertion and cough with no relieving factors. He reported gross painless hematuria throughout the urinary stream without clots. He is a chronic smoker with more than 40 pack years smoking history but denied alcohol and illicit drug use. He also denied fever, weight loss, night sweats, chills, hematemesis, melena, other genitourinary symptoms, incarceration, tuberculosis exposure, any recent travel, history of coagulopathy, and genetic disorders. Medication history included amlodipine for hypertension but no medication for COPD. The patient had no significant past surgical history and family history was noncontributory.

Pertinent findings during physical examination included the blood pressure of 135/80 and bilateral wheezing in the recumbent position. Rest of the physical examination was unremarkable.

The baseline investigations were unremarkable except serum creatinine at 1.81 mg/dl, blood urea nitrogen (BUN) 26 mg/dl with estimated glomerular filtration rate (EGFR) of 48.63 mL/min/1.73m². Urinalysis was positive for 3+ blood, 2+ protein, red blood cells (RBCs) >100/hpf and white blood cells (WBCs) 50-100/hpf. Urine sediment was positive for numerous dysmorphic RBCs. Chest X-ray showed emphysematous changes in lungs with no focal consolidation or pulmonary vascular congestion. Electrocardiogram (EKG) and transthoracic echocardiogram (TTE) did not reveal any abnormality. Computed tomography (CT) scan of the chest with intravenous (IV) contrast done in emergency department showed left lower lobe nodules (largest ~2x1 cm) with surrounding ground-glass opacities and general emphysematous changes. He was admitted for further evaluation of hemoptysis, hematuria, and lung nodule. During his course of hospital stay, serum creatinine increased to 3.12 mg/dl. C3 and C4 were within normal limit. Ultrasound showed normal-sized kidneys without hydronephrosis. Renal biopsy showed focal necrotizing glomerulonephritis with more than 40% cellular crescents. He was treated with IV methylprednisolone burst for three days and prednisone 60 mg daily.

**Figure 1:**
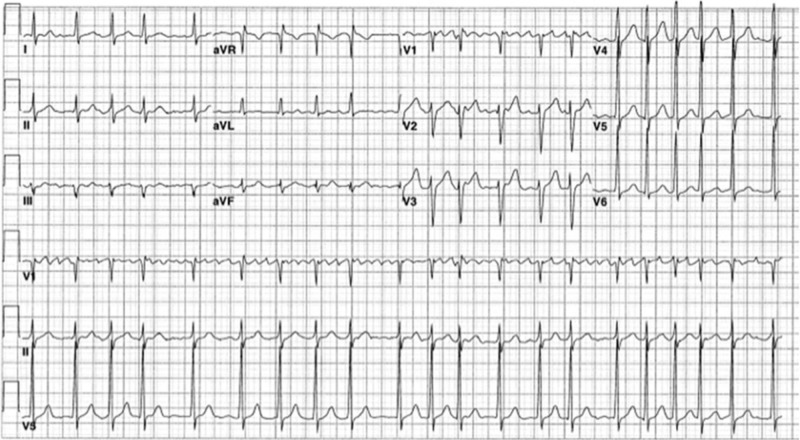
Electrocardiogram (EKG) showing atrial fibrillation with a rapid ventricular rate

Six days later, he received one dose of IV cyclophosphamide 793 mg. On the same day, he developed tachycardia at the rate of 130-140bpm (Figure 1). EKG showed tachycardia due to atrial fibrillation with rapid ventricular rate (RVR) that was initially treated with a Cardizem® drip in the medical intensive care unit (MICU). The patient reverted to sinus rhythm after 10-15 minutes of the treatment. TTE showed normal ejection fraction, trace regurgitation of mitral and tricuspid valves, but no vegetations. Anticoagulation was not started due to hemoptysis and hematuria (CHADS Vasc score=2).

The patient was transferred to the floor after weaning off the drip and transitioned on oral Cardizem® 240 mg daily. The patient was further evaluated to rule out other causes of atrial fibrillation including complete blood count (CBC), serum electrolytes, thyroid function tests, cardiac enzymes and chest X-ray, which were all within normal limits. During the rest of his hospital stay, the patient remained hemodynamically stable and did not develop another episode of atrial fibrillation. An echocardiogram was repeated before discharge which remained unchanged from his previous echocardiogram.

Given the associated onset of symptoms following the use of cyclophosphamide, normal investigations were performed to rule out other causes, and with no recurrence of atrial fibrillation following cessation of cyclophosphamide, the diagnosis of cyclophosphamide-induced atrial fibrillation was made.

## Discussion

Cyclophosphamide (CYA) is one of the few chemotherapeutic agents with the potential to cause cardiotoxicity. The spectrum of CYA-induced cardiac complications includes isolated tachyarrhythmias, myopericarditis, restrictive cardiomyopathy, cardiac tamponade, and fulminant cardiac failure [1]. The exact mechanism of its cardiotoxicity is not fully understood. The most likely mechanism of CYA-induced cardiac toxicity is direct endothelial damage by the drug itself and free radical injury by its metabolite, phosphamide, which induces endothelial and myocyte injury leading to extravasation of toxic metabolites and proteins [2-3]. The toxic metabolite causes apoptosis of myocardial cells and interstitial hemorrhage resulting in tachyarrhythmias and other cardiac complications.

The metabolism of CYA occurs in the liver by the P450 enzyme system, and the CYA metabolites produced are eliminated by kidneys. So patients with renal insufficiency are at greater risk of CYA-related cardiac toxicity even at lower doses. Thus, dose adjustment of CYA should be implemented to avoid its side effects. The high total dose of CYA per course is a well-known risk factor for cardiotoxicity, but different studies outline different thresholds [4]. Goldberg et al. suggested a threshold dose of approximately 1.5 g/m2 [5]. In this case, the patient developed atrial fibrillation with RVR after receiving a single dose of CYA that was 0.5 g/m2, well below the proposed threshold critical dose.

Through literature reviews, we noted a few other significant factors associated with increased risk of CYA-related cardiac toxicity. Advanced age and type of malignancy are among the eminent ones [6]. Greater risk of CYA-related cardiotoxicity was reported in patients with lymphoma as opposed to breast cancer. Moreover, prior radiation exposure to mediastinum and concomitant or prior use of other cardiotoxic agents increases the risk [7]. P450 enzyme inducers increase the risk of toxicity by increased production of cardiotoxic metabolites of CYA like phosphoramide and acrolein in the blood. Studies show that steroids like prednisone and dexamethasone are inducers of P450 [8]. In our case, the use of prednisone for more than five days most likely induced the P450 enzyme causing rapid accumulation of toxic metabolites of CYA and predisposing to cardiotoxicity.

The clinical manifestations in CYA-related cardiotoxicity are variable in severity and presentation depending on the specific pathology caused by CYA. Its toxicity typically manifests within the first 48 hours following the first dose but can occur up to 10 days later. Tachyarrhythmias like atrial fibrillation with RVR is an unusual complication associated with CYA use.

Early detection of CYA-related cardiotoxicity is mandatory due to its potential to cause rapidly progressive cardiac insult. Echocardiography and electrocardiography are fundamental for the initial evaluation of cardiotoxicity by chemotherapeutic agents. While EKG is helpful to rule out arrhythmias, the echocardiogram is not very reliable to detect early signs of CYA effects since tachyarrhythmias can mask evaluation of cardiac chambers and there can be a delay of reduction in left ventricular ejection fraction (LVEF). Cardiac magnetic resonance imaging (MRI) can be used to detect remodeling in heart chambers in the setting of high doses of CYA [9]. Cardiac markers like B-type natriuretic peptide (BNP) can be used to rule out acute cardiac failure especially if patients have received high doses. BNP is usually elevated by the second week so it is not an ideal marker for early diagnosis [10-11].

If there is any suspicion of CYA-related cardiotoxicity either clinically or by laboratory evidence, the most crucial step is to stop the drug immediately. Once diagnosed, the treatment of tachyarrhythmias is no different from the general approach. Medical treatment for rate control is used in hemodynamically stable patients. In cases of refractory arrhythmias or unstable patients, early recognition and involvement of coronary care unit or intensive care unit are imperative. Such patients require aggressive monitoring and hemodynamic support.

## Conclusions

All physicians should be aware of CYA-related cardiotoxicity because of its potentially devastating effects. Early detection of CYA-related cardiotoxicity is mandatory due to its potential to cause rapidly progressive cardiac insult. Most of the CYA-induced cardiac adverse effects occur within the first 48 hours after the initial dose. Dose adjustment of CYA should be implemented in patients who have advanced age, renal insufficiency, or concomitant use of prednisone and other cardiotoxic drugs.
